# High-fluence and high-gain multilayer focusing optics to enhance spatial resolution in femtosecond X-ray laser imaging

**DOI:** 10.1038/s41467-022-33014-4

**Published:** 2022-09-13

**Authors:** Hirokatsu Yumoto, Takahisa Koyama, Akihiro Suzuki, Yasumasa Joti, Yoshiya Niida, Kensuke Tono, Yoshitaka Bessho, Makina Yabashi, Yoshinori Nishino, Haruhiko Ohashi

**Affiliations:** 1 Japan Synchrotron Radiation Research Institute, 1-1-1, Kouto, Sayo-cho, Sayo-gun, Hyogo, 679-5198 Japan; 2 RIKEN SPring-8 Center, 1-1-1 Kouto, Sayo-cho, Sayo-gun, Hyogo, 679-5148 Japan; 3 Research Institute for Electronic Science, Hokkaido University, Kita 21 Nishi 10, Kita-ku, Sapporo, 001-0021 Japan; 4 Institute of Biological Chemistry, Academia Sinica, 128, Academia Road Sec. 2, Nankang, Taipei 115 Taiwan

**Keywords:** Imaging techniques, Imaging and sensing

## Abstract

With the emergence of X-ray free-electron lasers (XFELs), coherent diffractive imaging (CDI) has acquired a capability for single-particle imaging (SPI) of non-crystalline objects under non-cryogenic conditions. However, the single-shot spatial resolution is limited to ~5 nanometres primarily because of insufficient fluence. Here, we present a CDI technique whereby high resolution is achieved with very-high-fluence X-ray focusing using multilayer mirrors with nanometre precision. The optics can focus 4-keV XFEL down to 60 nm × 110 nm and realize a fluence of >3 × 10^5^ J cm^−2^ pulse^−1^ or >4 × 10^12^ photons μm^−2^ pulse^−1^ with a tenfold increase in the total gain compared to conventional optics due to the high demagnification. Further, the imaging of fixed-target metallic nanoparticles in solution attained an unprecedented 2-nm resolution in single-XFEL-pulse exposure. These findings can further expand the capabilities of SPI to explore the relationships between dynamic structures and functions of native biomolecular complexes.

## Abstract (web summary)

Here, the authors realize an ultra-high fluence X-ray laser by high-gain multilayer focusing optics. This enables in-solution imaging with 2-nm resolution in a single-pulse exposure, making strides toward biomolecular imaging under physiological conditions.

## Introduction

Atomic structure determination of non-crystalline specimens using X-ray free-electron laser (XFEL)^1^-based coherent diffractive imaging (CDI)^2,3^ has been envisaged by adopting the diffraction-before-destruction principle^4,5^ overcoming radiation damage limits^6,7^. Despite considerable efforts, the spatial resolution achieved using XFEL-based CDI is ~7 nm in 3D for a virus^8^, tens of nanometres for cell organelles^9^ and bacteria^10,11^, ~5.5 nm in 3D for symmetric metallic nanoparticles^12^, and <10 nm for other metallic nanoparticles^13,14^. More recently, gold nanoparticle ensembles were resolved at <3 nm^15^ in 3D by merging a large number of weak diffraction patterns with the help of the megahertz-rate XFEL, while using a micro-focus beam with a moderate photon density of 6 × 10^10^ photons μm^−2^ pulse^−1^.

There are obstacles that must be overcome to improve the single-shot spatial resolution^16,17^. In particular, insufficient photon fluence on samples presently restricts diffraction signals due to the weak interaction of X-rays with non-crystalline nanosized objects. So far, XFEL-based CDI of nanoparticles exploiting existing high-fluence focusing optics has not been able to experimentally produce diffraction patterns with sufficient signal and signal-to-noise levels to reconstruct structures reaching at 2-nm spatial resolution with single-shot femtosecond exposure. High-resolution observation is difficult because the scattered intensity decreases rapidly with increasing scattering vectors. More specifically, when assuming the inverse fourth power scaling with increasing scattering vector^18^, four orders of magnitude higher fluence is required to obtain an order of magnitude better spatial resolution. Focusing optics are key for enhancing the XFEL fluence to achieve a higher spatial resolution of femtosecond X-ray laser imaging.

Micro-/nano-focused XFEL pulses have been reported with diffractive^19,20^, reflective^15,21–23^, and refractive^24^ optics. Micro-focused total-reflection mirrors^21,23^ can routinely provide photon densities of >1 × 10^11^ photons μm^−2^ pulse^−1^. Some sophisticated total-reflection mirrors^22,23^ can produce higher fluences of >1 × 10^5^ J cm^−2^ pulse^−1^ or photon densities of >1 × 10^12^ photons μm^−2^ pulse^−1^. Total-reflection mirrors feature high efficiency and wide spectral acceptance compared to other diffractive zone plates and refractive lenses.

Higher demagnification optics can achieve higher fluence because a demagnified image of the XFEL source appears on the focal plane. The amount of demagnification is geometrically calculated as the source-to-optics distance divided by the focal length. Multilayer mirrors^20^ can surpass total-reflection mirrors in fluence by exploiting higher demagnifications with shorter focal lengths. They utilize the diffraction effect to achieve larger grazing-incidence angles and obtain larger spatial acceptances with shorter mirrors, thereby pushing the focal lengths shorter. The diffraction effect of multilayer mirrors nevertheless limits their use at specific photon energy. The development of multilayer focusing mirrors with large grazing-incidence angles, however, involves highly challenging manufacturing processes, including surface finishing and metrology of the steeply aspheric substrate surface.

In this study, we designed and established a CDI system to improve single-shot spatial resolution using high-fluence nano-precision XFEL-focusing multilayer mirrors by developing techniques for multilayer deposition, ultra-precision surface processing^25^, and metrology. The system allows us to collect diffraction signals up to a spatial frequency of 0.50 nm^−1^ (2 nm spatial resolution). Nanoscale observations of the structure and dynamics of nanoparticles in their native environment will tremendously contribute to the understanding of their functions. For solution samples, micro-liquid enclosure arrays (MLEAs)^10^ can be utilized in fixed-target CDI measurements, as shown later.

## Results

### Design and fabrication of focusing optics

In designing high-fluence multilayer focusing optics, we set the targeted focal spot size to be around 100 nm^17^ by considering that the spot size should be larger than the sample particle. For high-resolution CDI, it is also important to have enough working distance to install guard slits right before the sample to suppress parasitic background scattering from the multilayers and other upstream optics. In measuring extremely weak scattering signals from non-crystalline nanometre-sized objects, reducing background scattering is as important as increasing fluence.

**Fig. 1:**
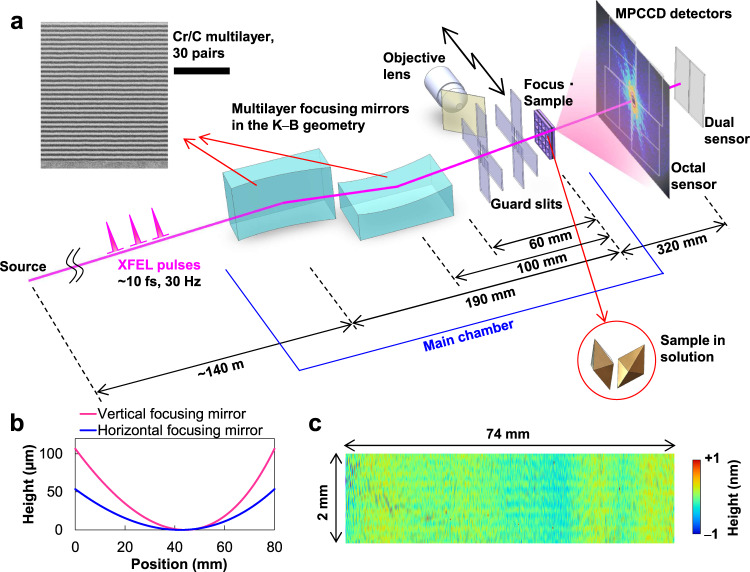
Experimental setup of the developed coherent diffractive imaging (CDI) system. **a** Schematic layout. X-ray free-electron laser (XFEL) pulses were focused by multilayer mirrors in the Kirkpatrick–Baez (K–B) geometry. Scattered X-rays from sample particles at the focus were recorded with multiport charge-coupled device (MPCCD) detector system. The top left inset shows the cross-sectional scanning transmission electron microscope (STEM) image of (Cr/C)_30_ multilayer employed in this optical system; the scale bar is 60 nm. **b** Surface shapes of the designed mirror substrates. **c** Measured surface figure error of the horizontal focusing mirror.

We employed elliptical mirrors in the Kirkpatrick–Baez (K–B) geometry^26^ utilizing Cr/C multilayers with a large spatial acceptance of 1850 μm square, which is enough to reflect most of an incident XFEL beam with a full width at half-maximum (FWHM) of ~550 μm. Figure 1a shows the experimental setup of the developed CDI system. The mirror substrates have steeply curved elliptical surfaces as shown in Fig. 1b with a radius of curvature from 4 to 20 m. Table 1 lists the optical parameters, which were optimized for CDI experiments to achieve the best possible fluence in a 100 nm focus with a photon energy of 4 keV (wavelength of 0.31 nm) at an XFEL facility of the SPring-8 Angstrom Compact free electron LAser (SACLA)^27^. (See Methods for the design of the focusing optics.) The designed focus sizes were ~110 nm and ~60 nm (FWHM) in the horizontal and vertical directions, respectively, principally determined by the geometric demagnification of the XFEL source size of ~80 μm (FWHM). (See ref. 21 for more detail about a method for estimating an ideal focus size of an XFEL.) The diffraction-limited focus sizes of the multilayer optics were 94 nm and 50 nm (FWHM) at 550 μm square apertures in the horizontal and vertical directions, respectively, which are slightly smaller than the design sizes. Here, it is noteworthy that focusing optics having a shorter focal length and a larger numerical aperture help reduce the blurring due to diffraction. Furthermore, a shorter focal length of the mirror optics leads to the suppression of the positional displacement of the focused beam accompanied by an incident angle error caused by such as environmental vibrations. For example, when a focal length is as wide as 1 m, even a small incident-angle error of 0.05 μrad causes a 100-nm displacement in the focal position, which is considerably large for a 100-nm scale focusing. Here, the deviation angle between the incident ray and reflected ray is twice the glancing angle. The high positional stability of the focused beam is advantageous in aligning the optics and the samples and contributes to suppress the background noise scattering.

**Table 1:** Optical parameters of designed focusing optics

	Horizontal focusing mirror	Vertical focusing mirror
Surface profile	Elliptical cylinder	Elliptical cylinder
Substrate material	Synthetic fused silica	Synthetic fused silica
Effective mirror length	74 mm	74 mm
Mirror substrate size	80 mm × 50 mm × 30 mm (thickness)	80 mm × 50 mm × 30 mm (thickness)
Glancing angle on optical axis	25 mrad	25 mrad
Focal length	190 mm	100 mm
Semimajor axis	70.095 m	70.095 m
Semiminor axis	128.9245 mm	93.56175 mm
Spatial acceptance	1850 μm	1850 μm
Surface coating	(Cr/C)_30_ multilayer	(Cr/C)_30_ multilayer
Focal spot size	~110 nm (FWHM)	~60 nm (FWHM)
Depth of focus (Twice the Rayleigh range)	±40 µm	±10 µm

We developed multilayer X-ray focusing mirrors with nanometre precision using ultra-precision fabrication technologies, including surface processing^25^ and metrology, which is the most difficult technical hurdle in advanced fabrication (See Methods for more detail about the mirror fabrication). To produce the ideal focus with the designed beam sizes and low intensities outside the main peak, the figure errors and roughnesses of the substrate surfaces were reduced to 0.18 nm (0.23 nm) (evaluation area: 74 mm × 2.0 mm) (Fig. 1c) and 0.20 nm (0.46 nm) (evaluation area: 282 μm × 212 μm), respectively, in terms of the root mean square (r.m.s.) for the horizontal (vertical) focusing mirrors. The evaluation results fall within the criterion of the Rayleigh quarter-wavelength rule^28^ for a figure error height of 1.55 nm (peak-to-valley) (~0.4 nm (r.m.s.)) and provide specular reflectivity of >0.8, as calculated from the Debye–Waller factor at a surface roughness of 0.47 nm (r.m.s.).

### Characterization of focusing optics

**Fig. 2:**
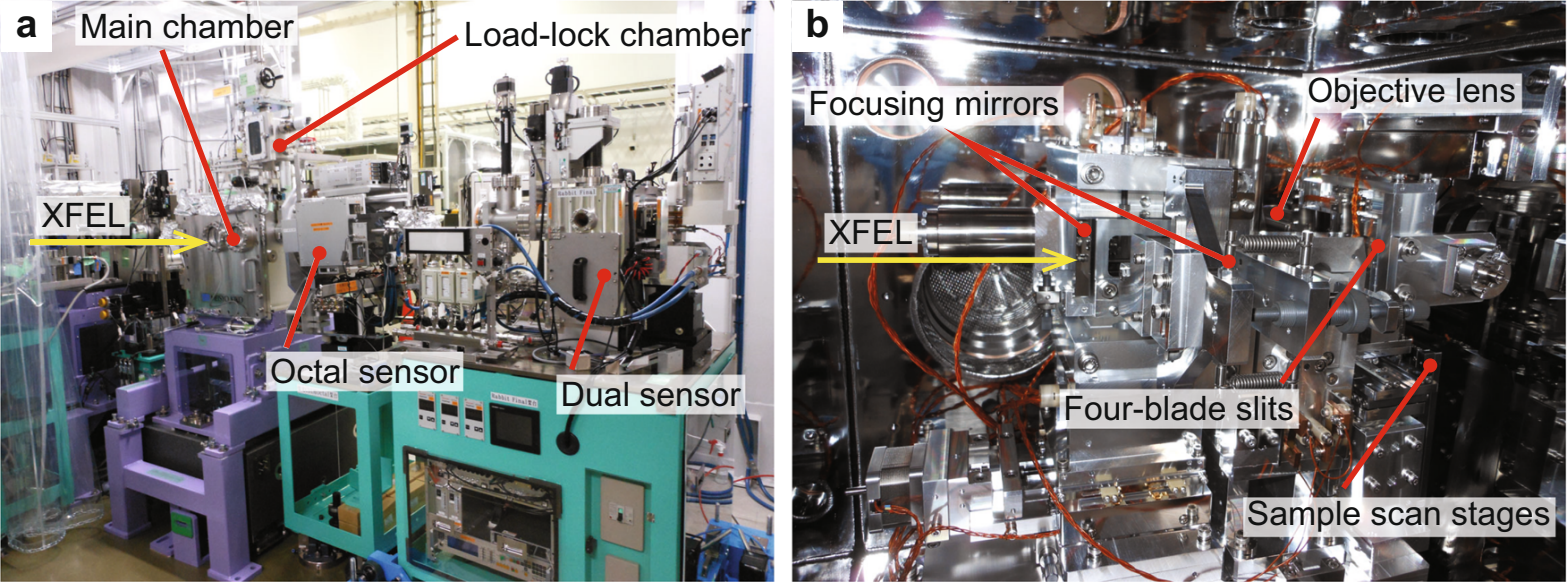
Photograph of the experimental apparatus for the CDI system at EH3 BL2 of the SPring-8 Angstrom Compact free electron Laser (SACLA). **a** The main chamber, which contains (**b**) the focusing optics with a mirror manipulator and sample scan stages, is connected to the MPCCD detector system. In the distance of 60 mm between the downstream edge of the multilayer X-ray focusing optics and the focus, the system has two sets of four-blade slits (guard slits) and an objective lens with a 90° reflection mirror, which are switchable using a linear translation stage. The four-blade slits composed of tapered Si are essential for the CDI measurement to suppress unwanted background (parasitic) scattering from the upstream X-ray optics, including the multilayer mirrors. The optical microscope utilizes the objective lens to observe the sample at the focus using an imaging sensor located on the side of the main chamber. Samples are loaded from a load-lock chamber using a sample transfer system. Turbomolecular pumps can evacuate the main chamber to a pressure of <1 × 10^−4^ Pa.

The entire CDI instrument utilizing multilayer X-ray focusing mirrors is referred to as MAXIC-S. As shown in Fig. 2, the MAXIC-S main chamber includes compactly arranged components (focusing optics with a mirror manipulator, three-axis sample scanning stages, etc.) and is combined with a multiport charge-coupled device (MPCCD) detector^29^ system that collects the diffraction signals from objects. The best achievable full-period resolution is ~2.0 nm at the edge of the MPCCD detector.

**Fig. 3:**
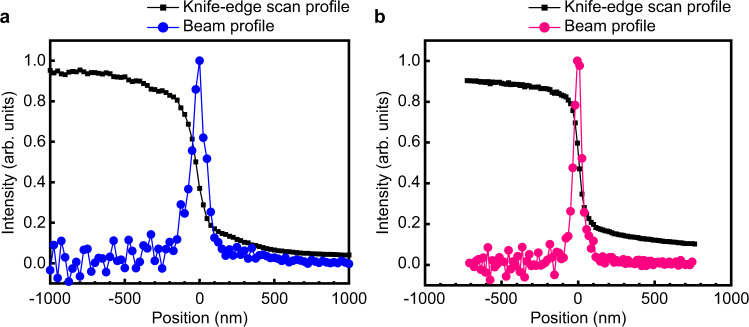
Evaluated intensity distributions of focused beam. The intensity distributions in the focal plane were measured using a knife-edge scanning method at an XFEL repetition rate of 30 Hz. The evaluated focal spot size was **a** 110 nm (full width at half-maximum (FWHM)) in the horizontal direction and **b** 60 nm (FWHM) in the vertical direction at scan steps of 25 and 15 nm, respectively. In **a** and **b**, intensities of 600 and 100 XFEL pulses were averaged for each data point, respectively.

We evaluated the focused beam performance at BL2 of SACLA. We used the knife-edge scanning method to obtain intensity distributions in the horizontal and vertical directions for simplicity. For single-shot characterization of two-dimensional intensity distributions near the focus, wavefront sensors^30,31^ are suitable. Figure 3 shows the intensity distributions in the focal plane measured by a knife-edge scanning method. (See Methods for more detail about the evaluations of the focused beam properties.) We observed a focused beam with dimensions of 110 nm × 60 nm (FWHM) and a reflectivity of 52% at double reflections. These results are almost consistent with the designed focus dimensions and theoretical reflectivity of 62% calculated at an interdiffusion/roughness of 0.3 nm (r.m.s.). The knife-edge scan shows that 50% of the reflected X-rays are focused in ranges of 142 nm and 86 nm in the horizontal and vertical directions, respectively. With these beam sizes and evaluated pulse energy of 61 μJ (9 × 10^10^ photons) at the focus, the fluence at the focus was estimated to be 3 × 10^5^ J cm^−2^ pulse^−1^, that is, the photon density was 4 × 10^12^ photons μm^−2^ pulse^−1^, and the intensity was 3 × 10^19^ W cm^−2^, assuming a pulse duration of 10 fs^32,33^. The above evaluation was performed for an average XFEL pulse energy of 470 μJ just after the undulator^34^. When thermally stabilized about two days after the initial alignment, the best focus sizes can be maintained for 24 h or longer.

### Demonstration of femtosecond X-ray laser imaging

**Fig. 4:**
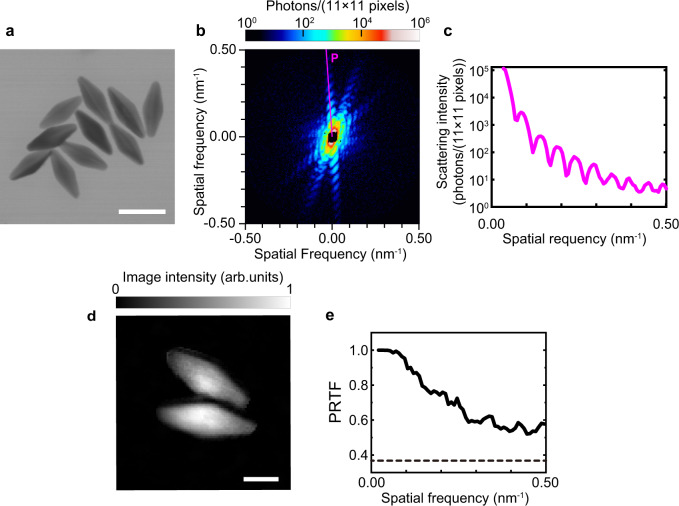
Single-shot coherent X-ray diffraction (CXD) pattern and image reconstruction. **a** STEM image of bipyramidal Au nanoparticles (AuNPs) with a width and length of ~20 nm and ~55 nm, respectively. The scale bar is 50 nm. **b** Measured CXD pattern from bipyramidal AuNPs binned by 11 × 11 pixels. **c** Profile along line P in **b**. **d** Reconstructed image of two bipyramidal AuNPs with a pixel size of 1 nm. The image is normalized to a 0–1 scale. The scale bar is 20 nm. **e** Phase-retrieval transfer function (PRTF) for the reconstructed image shown in **d**. The resolution was estimated to be 2.0 nm, based on the criterion of a PRTF threshold of 1/*e*.

To demonstrate CDI measurement with MAXIC-S, we measured bipyramidal Au nanoparticles (AuNPs) (from Nanoseedz Ltd.) in distilled water containing <0.02 wt% cetyltrimethylammonium bromide (CTAB). Bipyramidal AuNPs with controlled size and shape are available due to well-established synthetic procedures. Nanoparticles generally possess unique physical and chemical properties depending on their shapes and sizes, and can find a wide range of applications^35^. Bipyramidal AuNPs are anticipated to improve various plasmonic devices owing to their high tunability and optical sensitivity in solution^36^. Figure 4a shows a scanning transmission electron microscope image of the sample AuNPs, which indicates the well-known controlled shape. Each AuNP has a size of ~20 nm in width and ~55 nm in length, and its volume is as small as a ~20-nm cube. The sample solution in an MLEA was installed at the focal point of the multilayer focusing mirrors. Figure 4b depicts the single-shot coherent X-ray diffraction (CXD) pattern of a bipyramidal AuNP object. The central black area is the open aperture of the MPCCD octal-sensor detector. Figure 4c presents the profile along line P in the CXD pattern of Fig. 4b. The guard slits set at a short distance of about 2 mm from their downstream end to the focus effectively reduced the background noise scattering on the MPCCD detector system. High-quality diffraction signals with practically low scattered backgrounds were recorded in a spatial frequency region ranging from 0.021 to 0.50 nm^−1^ (near the edge of the detector). The background noise scattering from the upstream optics, including the multilayer X-ray focusing mirrors, was three orders of magnitude smaller than the signal from the AuNPs even at the detector edge. Figure 4d shows the sample image reconstructed by iterative phase-retrieval processes. (See Methods for more details about the data processing and image reconstruction.) The two bipyramidal AuNPs with lengths of 58 and 55 nm and a common width of 21 nm were clearly reconstructed, showing that the image is consistent with the expected morphology. The full-period resolution is 2 nm (Fig. 4e) estimated from the phase-retrieval transfer-function^37^, which measures the fidelity of the retrieved phases and is typically used to assess the resolution of the reconstructions.

## Discussion

The developed CDI optics have a distinct advantage in that the fluence at the focus is 4 × 10^4^ times higher than that at the light source position. This total gain (the fluence ratio) measures the focusing performance of the optical system throughout the entire beamline, including the loss by the transmissions, reflectivities, and apertures of all the optical elements in the optical path. The total gain is more than tenfold higher than the other existing optics for XFEL-based CDI^38,39^. Specifically, it is around tenfold higher than the 100 nm focusing at Linac Coherent Light Source (LCLS)^23^ and European XFEL (designed)^40^, and much more than a hundredfold higher than the μm-scale focusing at European XFEL^15^ (See Supplementary Table 1). Here, the tenfold enhancement in the total gain and potential fluence corresponds to about a twofold improvement in the spatial resolution when assuming the inverse fourth power scaling of scattered intensities with increasing scattering vector. Despite the relatively low pulse energy of SACLA (470 μJ), which is about one order of magnitude weaker than that of the LCLS^1^ and the European XFEL^41^, the extremely high fluence at the sample position realized by the high-gain multilayer focusing mirrors enabled XFEL-based CDI with an unprecedented high 2-nm resolution by single-shot femtosecond observation.

The total number of electrons in the sample particles can be used to infer the strength of X-ray scattering because X-rays are mainly scattered by electrons. From the particle sizes of the reconstructed image and density of Au, we estimated the total number of electrons in the two bipyramidal AuNPs to be ~8.5 × 10^7^. This value is of the same order of magnitude as that of, for example, a single rice dwarf virus particle of ~70 nm in diameter^42^. It is significant that nanoparticles with low X-ray scattering power comparable to a virus particle were imaged in solution at 2-nm spatial resolution by a single femtosecond XFEL pulse exposure. Note that the X-ray scattering is weaker for particles in solution than in a vacuum because the electron density difference between the particles and solvent contributes to the X-ray scattering. The developed CDI system will provide valuable technical expertise contributing directly to the future observation of biological nanoparticles in solution. Further studies are needed to image biological nanoparticles such as viruses, because scattering intensities depend on the structure factor in addition to the number of electrons in an object at a given incident fluence. The larger electron-density difference between the gold particles and solvent results in higher scattering intensities compared to viruses, even though the total number of electrons of the objects is similar (See Supplementary Note 1 and Supplementary Figs. 1–3).

The scattering intensity at zero momentum transfer is proportional to the incident X-ray fluence and the square of the total number of electrons in the sample. The estimated total number of electrons in the sample particles and the solvent electron density can also be used to evaluate the fluence of the focused XFEL pulse based on the total intensity of the reconstructed image. A recovered scattering intensity at zero momentum transfer was used to estimate the fluence. We compared calculated and measured CXD patterns to validate the incident fluence (See Supplementary Fig. 4). Similar calculations to estimate an incident fluence were conducted in previous studies^15,23^. The evaluated photon density of 9.1 × 10^12^ photons μm^−2^ pulse^−1^, which is approximately twofold greater than that determined by the knife-edge evaluation, validates the reliability of the evaluated fluence. The results are acceptable because the knife-edge scans were performed by averaging multiple shots, and the pulse-to-pulse fluctuations in the position and profile of the XFEL can cause underestimation of the fluence. Nevertheless, the focused beam was stable regarding the focused beam position and size enough to measure with the knife-edge scan method by taking advantage of the short focal lengths.

The evaluated fluence reached a level almost comparable to those used in realistic single-particle imaging (SPI) simulations for biomolecules in 3D with sub-nanometre resolution^40,43^. SPI in 3D, though the image is an average of many particles, can offer high resolution by merging many diffraction patterns from reproducible particles, even if each diffraction pattern is so weak that it is impossible to reconstruct a 2D image by itself. It is noteworthy that single-shot X-ray laser imaging in 2D can characterize the distinct structure of individual nanoparticles and can provide an insight into dynamic nature at a temporal resolution of around ten femtoseconds.

Highly efficient ellipsoidal multilayer mirrors^44^ may further increase the fluence with ~100 nm focusing by twofold or more by using identical horizontal and vertical focal spot sizes and enhancing the reflectivity with a single reflection, although further technical developments are necessary. In addition, it is desired to increase the focusing fluence by improving XFEL sources in terms of the pulse energy and incident XFEL beam profile. High-fluence XFEL-based CDI will contribute to the exploration of the dynamic properties of native biological specimens in solution at physiological temperatures and will be complementary to cryo-electron microscopy.

## Methods

### Design of focusing optics

The materials of the multilayer mirrors, Cr and C, were determined to obtain high reflectivity for 4 keV XFELs with a broad reflection bandwidth at >100 eV (FWHM), which is sufficiently larger than the ~30 eV (FWHM) spectral bandwidth of the incident XFEL beam measured at BL2 of SACLA. We evaluated the radiation doses absorbed under intense XFEL radiation by the multilayer materials composed of Cr and C and made the dose adequately below the damage threshold level. The incident photon energy of 4 keV is the lowest photon energy available at the beamline and was selected to obtain high X-ray diffraction signals by considering the energy dependences of the following factors: (1) the pulse energy at the XFEL source (~500 (~600) μJ or ~8 × 10^11^ (~4 × 10^11^) photons at a photon energy of 4 (10) keV), (2) the transmission of the optical elements in the optical path (a beam intensity/position monitor with a nanocrystalline chemical vapour deposition diamond scatterer^34^ (15 μm thick; transmission is 83% at a photon energy of 4 keV) and two Be windows (60 μm thick each; the transmission is 91% each at a photon energy of 4 keV)), (3) the reflectivities (82% at a photon energy of 4 keV) of the two 600-mm-long Si plane mirrors placed in the optics hutch with a glancing angle of 4 mrad to remove γ-rays, (4) the coherent scattering cross-sections of the object materials^6^, and (5) the detection efficiency of the MPCCD detector to collect diffraction signals from objects (See Supplementary Fig. 5 for more details about the energy dependences of efficiency for various factors).

### Mirror fabrication

The substrate surfaces of the multilayer mirrors were initially formed to be elliptical–cylindrical with an accuracy of <50 nm (peak-to-valley) using a conventional machining process and polishing method. Then, the surface roughness of the mirror substrates was removed to suppress background noise scattering using elastic emission machining (EEM)^45^, an ultra-precision surface processing method, with a rotary-type working head. After that, the substrate surfaces were finished using a computer-controlled figure-correction method^25^ based on nozzle-type EEM. In this deterministic figuring process, we utilized the metrology system combined with a stitching interferometer (SI) and scanning probe profilometer (a zero-method scanning-probe profilometer (ZSP)), which were developed for strongly curved X-ray mirrors by our group, to achieve high-precision measurement. The measurement reproducibility for the SI is <0.1 nm (r.m.s.) in the spatial frequency range from 100 μm to ~10 mm, and that for the ZSP is ~0.2 nm (r.m.s.) at spatial frequencies of more than 10 mm. The surface profiles of the mirror substrates were evaluated at a measurement precision of ~0.2 nm (r.m.s.) by merging the measured profiles with the SI and ZSP (See Supplementary Fig. 6 for measurement results of the SI and ZSP). After finishing the mirror substrates, laterally graded (Cr/C)_30_ multilayers with a period of 6.8 nm at the mirror centre were coated using a DC magnetron sputtering method. The ratio of (Cr layer thickness)/(Period) is 0.5 (design). The top material is C. The bottom material is Cr. No bonding layer is used (See Supplementary Fig. 7 for the lateral profiles and the reflectivity of (Cr/C)_30_ multilayer).

### Evaluations of focused beam properties

We performed precision optical alignment of the focusing mirrors based on the alignment tolerances estimated by conducting wave-optical focusing simulations. A Foucault knife-edge test with 200-μm-diameter Au wires was performed to optimize the incident angles, perpendicularity between mirrors, and focal lengths. To avoid melting and ablation of the wire by the intensely focused XFEL beam, the incident XFEL beam was attenuated while maintaining the focal spot sizes by using polyimide films and single-crystal Si plates with mirror-polished surfaces with total thicknesses of 250 and 90 μm, respectively. The third-order harmonic contamination at 12 keV is negligibly small relative to the first-order harmonic at 4 keV, and is estimated to be of the order of 10^−9^ (See Supplementary Figs. 5 and 7c for the reflectivity and transmission of the optical components). The focal planes for horizontal and vertical focusing were adjusted to be coplanar with a precision of 5 μm by performing a Foucault knife-edge test with 10-μm-diameter Au wires and observing the horizontal and vertical wires via an optical microscope, which had an objective with a numerical aperture of 0.3, corresponding to a depth of focus of 6 μm. The depth of focus of the focused XFEL beam was more than ±18 μm and ±5 μm within which the beam size calculated based on geometric optics using the incident beam size is <110 nm and 60 nm in the horizontal and vertical directions, respectively. The centre of the incident photon energy was tuned at 4.05 keV to obtain the optimum reflectivity of the multilayer mirrors. This value corresponds to a 1.0% thickness error of the multilayers. We measured the intensity distributions of the focused beam using a 200-μm-diameter Au wire as a knife-edge. Stick-slip piezo drive stages (SLC-2490-D-S-HV, SmarAct GmbH) were used as scanning stages of the measurements at a positioning resolution of 5 nm by closed-loop position control. We normalized the incident pulse energy measured with an intensity monitor^34^ to eliminate the shot-to-shot intensity fluctuation of the XFEL pulses. Fluctuation of pulse energy generated at the source was typically around 10% (r.m.s.) in 30 shots^46^. During the measurements, a four-blade slit upstream of the focusing mirrors limited the spatial acceptance of the focusing mirrors to 840 and 600 μm in the horizontal and vertical directions, respectively, which was the same condition as in the CDI experiments. Meanwhile, the measured incident beam sizes were ~570 and 540 μm (FWHM) in the horizontal and vertical directions, respectively. Consequently, the intensities outside of the main peak of the focus were removed to decrease the background noise scattering on the MPCCD detector.

### Data processing and image reconstruction

Two different types of MPCCD detectors, octal-sensor and dual-sensor detectors, both having a pixel size of 50 μm × 50 μm, were arranged in tandem. The MPCCD octal-sensor detector had a variable-opening aperture at the centre and was positioned 0.32 m downstream from the focus. The MPCCD dual-sensor detector was placed at 1.60 m downstream from the focus and collected diffracted X-rays at small angles, which passed through the central clear aperture of the octal-sensor detector. Just upstream from the dual sensor was a beam stop. The MPCCD octal-sensor detector had 2048 × 2048 pixels, whereas the dual-sensor detector had 1024 × 1024 pixels. Single-shot CXD patterns from bipyramidal AuNPs were recorded by the MPCCD octal sensor with a central aperture of 5.0 mm. The data in the central aperture and the gaps between the sensor panels were masked out. The highest spatial frequency of the CXD pattern used for data processing was 0.5 nm^−1^, which almost corresponded to the edge of the octal sensor. The pixel size of the reconstructed images was thus 1 nm. Image reconstruction was performed in two steps on the CXD pattern after electronic noise subtraction, centrosymmetrization, and binning by 11 × 11 pixels. The first step was to define the support area using the relaxed averaged alternating reflection (RAAR) algorithm^47^ coupled with shrink-wrap^48^ (8000 steps) followed by 100 iterations using the error reduction (ER) algorithm^49^. The initial support was created from the Patterson function (the autocorrelation of the sample). The feedback parameter *β* of the RAAR algorithm was set to 0.90, and the support was updated every 100 iterations with a threshold of 0.08. We initially set the kernel of the shrink-wrap algorithm to 5 pixels and gradually reduced it to 3 pixels. Using the reconstructed images with 10,000 different initial random seeds, the correlation coefficients (c.c.) were calculated between all pairs of images. The images were classified into several groups by using the c.c., and the support for the second step was defined by the majority. In the second step, the reconstruction consisted of 8,000 iterations with the RAAR algorithm followed by 100 iterations with the ER algorithm with the fixed support. The feedback parameter *β* of the RAAR algorithm was set to 0.90, as in the first step. Using the results with 10,000 different initial random seeds, 991 images with high similarity (c.c. > 0.995) were selected and averaged.

## Supplementary information

Supplementary Information

## Data Availability

The data that support the findings of this study can be found in the article and the Supplementary Information file. Any other relevant data are available from the corresponding author upon request.

## References

[1] Emma P (2010). First lasing and operation of an ångstrom-wavelength free-electron laser. Nat. Photonics.

[2] Chapman HN (2006). Femtosecond diffractive imaging with a soft-X-ray free-electron laser. Nat. Phys..

[3] Miao J, Ishikawa T, Robinson IK, Murnane MM (2015). Beyond crystallography: Diffractive imaging using coherent x-ray light sources. Science.

[4] Neutze R, Wouts R, Van der Spoel D, Weckert E, Hajdu J (2000). Potential for biomolecular imaging with femtosecond X-ray pulses. Nature.

[5] Gaffney KJ, Chapman HN (2007). Imaging atomic structure and dynamics with ultrafast X-ray scattering. Science.

[6] Shen Q, Bazarov I, Thibault P (2004). Diffractive imaging of nonperiodic materials with future coherent X-ray sources. J. Synchrotron Radiat..

[7] Howells MR (2009). An assessment of the resolution limitation due to radiation-damage in X-ray diffraction microscopy. J. Electron. Spectrosc. Relat. Phenom..

[8] Assalauova D (2020). An advanced workflow for single-particle imaging with the limited data at an X-ray free-electron laser. IUCrJ.

[9] Hantke MF (2014). High-throughput imaging of heterogeneous cell organelles with an X-ray laser. Nat. Photonics.

[10] Kimura T (2014). Imaging live cell in micro-liquid enclosure by X-ray laser diffraction. Nat. Commun..

[11] Van Der Schot G (2015). Imaging single cells in a beam of live cyanobacteria with an X-ray laser. Nat. Commun..

[12] Xu R (2014). Single-shot three-dimensional structure determination of nanocrystals with femtosecond X-ray free-electron laser pulses. Nat. Commun..

[13] Takahashi Y (2013). Coherent diffraction imaging analysis of shape-controlled nanoparticles with focused hard X-ray free-electron laser pulses. Nano Lett..

[14] Ihm Y (2019). Direct observation of picosecond melting and disintegration of metallic nanoparticles. Nat. Commun..

[15] Ayyer K (2021). 3D diffractive imaging of nanoparticle ensembles using an x-ray laser. Optica.

[16] Sun Z, Fan J, Li H, Jiang H (2018). Current status of single particle imaging with X-ray lasers. Appl. Sci..

[17] Aquila A (2015). The linac coherent light source single particle imaging road map. Struct. Dyn..

[18] Starodub D (2008). Dose, exposure time and resolution in serial X-ray crystallography. J. Synchrotron Radiat..

[19] David C (2011). Nanofocusing of hard X-ray free electron laser pulses using diamond based Fresnel zone plates. Sci. Rep..

[20] Matsuyama S (2018). Nanofocusing of X-ray free-electron laser using wavefront-corrected multilayer focusing mirrors. Sci. Rep..

[21] Yumoto H (2013). Focusing of X-ray free-electron laser pulses with reflective optics. Nat. Photonics.

[22] Mimura H (2014). Generation of 10^20^ W cm^−2^ hard X-ray laser pulses with two-stage reflective focusing system. Nat. Commun..

[23] Daurer BJ (2017). Experimental strategies for imaging bioparticles with femtosecond hard X-ray pulses. IUCrJ.

[24] Schropp A (2013). Full spatial characterization of a nanofocused x-ray free-electron laser beam by ptychographic imaging. Sci. Rep..

[25] Yumoto H, Koyama T, Matsuyama S, Yamauchi K, Ohashi H (2014). Ultra-high-precision surface processing techniques for nanofocusing ellipsoidal mirrors in hard X-ray region. in. Proc. SPIE.

[26] Kirkpatrick P, Baez AV (1948). Formation of optical images by X-rays. J. Opt. Soc. Am..

[27] Ishikawa T (2012). A compact X-ray free-electron laser emitting in the sub-ångström region. Nat. Photonics.

[28] Born, M. & Wolf, E. *Principles of Optics: Electromagnetic Theory Of Propagation, Interference and Diffraction of Light*, 7th edn. (Cambridge Univ. Press, Cambridge, 2006).

[29] Kameshima T (2014). Development of an X-ray pixel detector with multi-port charge-coupled device for X-ray free-electron laser experiments. Rev. Sci. Instrum..

[30] Seaberg MH (2019). Nanofocus characterization at the Coherent X-ray Imaging instrument using 2D single grating interferometry. Proc. SPIE.

[31] Makita M (2020). Double grating shearing interferometry for X-ray free-electron laser beams. Optica.

[32] Inubushi Y (2017). Measurement of the X-ray spectrum of a free electron laser with a wide-range high-resolution single-shot spectrometer. Appl. Sci..

[33] Inoue I, Tamasaku K, Osaka T, Inubushi Y, Yabashi M (2019). Determination of X-ray pulse duration via intensity correlation measurements of X-ray fluorescence. J. Synchrotron Radiat..

[34] Tono K (2011). Single-shot beam-position monitor for x-ray free electron laser. Rev. Sci. Instrum..

[35] Burda C, Chen X, Narayanan R, El-Sayed MA (2005). Chemistry and properties of nanocrystals of different shapes. Chem. Rev..

[36] Lee J-H, Gibson KJ, Chen G, Weizmann Y (2015). Bipyramid-templated synthesis of monodisperse anisotropic gold nanocrystals. Nat. Commun..

[37] Chapman HN (2006). High-resolution ab initio three-dimensional x-ray diffraction microscopy. J. Opt. Soc. Am. A.

[38] Boutet S, Williams GJ (2010). The coherent X-ray imaging (CXI) instrument at the Linac Coherent Light Source (LCLS). N. J. Phys..

[39] Bean RJ, Aquila A, Samoylova L, Mancuso AP (2016). Design of the mirror optical systems for coherent diffractive imaging at the SPB/SFX instrument of the European XFEL. J. Opt..

[40] Yoon CH (2016). A comprehensive simulation framework for imaging single particles and biomolecules at the European X-ray Free-Electron Laser. Sci. Rep..

[41] Decking W (2020). A MHz-repetition-rate hard X-ray free-electron laser driven by a superconducting linear accelerator. Nat. Photonics.

[42] Munke A (2016). Coherent diffraction of single Rice Dwarf virus particles using hard X-rays at the Linac Coherent Light Source. Sci. Data.

[43] Fortmann-Grote C (2017). Start-to-end simulation of single-particle imaging using ultra-short pulses at the European X-ray Free-Electron Laser. IUCrJ.

[44] Yumoto H (2017). Ellipsoidal mirror for two-dimensional 100-nm focusing in hard X-ray region. Sci. Rep..

[45] Yamauchi K, Mimura H, Inagaki K, Mori Y (2002). Figuring with subnanometer-level accuracy by numerically controlled elastic emission machining. Rev. Sci. Instrum..

[46] Tono K, Hara T, Yabashi M, Tanaka H (2019). Multiple-beamline operation of SACLA. J. Synchrotron Radiat..

[47] Luke DR (2004). Relaxed averaged alternating reflections for diffraction imaging. Inverse Probl..

[48] Marchesini S (2003). X-ray image reconstruction from a diffraction pattern alone. Phys. Rev. B.

[49] Gerchberg RW, Saxton WO (1972). A practical algorithm for the determination of phase from image and diffraction plane pictures. Optik.

