# Supplementary material for: High-fluence and high-gain multilayer focusing optics to enhance spatial resolution in femtosecond X-ray laser imaging
Source: Nat Commun. 2022 Sep 13;13:5300. doi: 10.1038/s41467-022-33014-4 (PMC9470745; doi:10.1038/s41467-022-33014-4)
Supplement: Supplementary file 1 — Supplementary Information [file 41467_2022_33014_MOESM1_ESM.pdf]

Supplementary Information for

High-fluence and high-gain multilayer focusing optics to enhance spatial resolution in femtosecond X-ray laser imaging

Hirokatsu Yumoto[1,2]*, Takahisa Koyama[1,2], Akihiro Suzuki[3], Yasumasa Joti[1,2], Yoshiya Niida[3], Kensuke Tono[1,2], Yoshitaka Bessho[2,4], Makina Yabashi[1,2], Yoshinori Nishino[3*], and Haruhiko Ohashi[1,2]

[1] Japan Synchrotron Radiation Research Institute, 1-1-1, Kouto, Sayo-cho, Sayo-gun, Hyogo 679-5198, Japan.

[2] RIKEN SPring-8 Center, 1-1-1 Kouto, Sayo-cho, Sayo-gun, Hyogo 679-5148, Japan.

[3] Research Institute for Electronic Science, Hokkaido University, Kita 21 Nishi 10, Kita-ku, Sapporo 001-0021, Japan.

[4] Institute of Biological Chemistry, Academia Sinica, 128, Academia Road Sec. 2, Nankang, Taipei 115, Taiwan.

*e-mail: yumoto@spring8.or.jp, yoshinori.nishino@es.hokudai.ac.jp

Supplementary Table 1| Optical parameters of the focusing mirrors in coherent diffractive imaging (CDI) used for LCLS, European-XFEL, and SACLA.

		**CXI LCLS[1] 100 nm focusing K–B mirrors**			**SPB/SFX European-XFEL[5]**		**MAXIC-S SACLA**
					**µm-scale focusing**	**100 nm scale focusing**	
		**CDI**	**Focus characterization**		**CDI**	**CDI**	**CDI**
		**Measured[2]**	**Wavefront assessment[3]**	**Wavefront assessment[4]**	**Measured[6]**	**Design and calculation[7]**	**Measured**
**Focal lengths:** (Design)	Source to mirror centre	~440 m			894.779 m, 896.459 m	915.484 m, 916.584 m	140 m, 140.19 m
	Mirror centre to focus	0.9 m, 0.5 m	(Same as on the left)	(Same as on the left)	24.005 m, 22.325 m	3.3 m, 2.2 m	0.19 m, 0.10 m
**Demagnification ratio**		~1/489, ~1/880			~1/37.3, ~1/40.2	~1/277.4, ~1/416.6	~1/736.8, ~1/1401.9
**Focus:**	Size (FWHM)	522 nm (*1)	**118 nm × 81 nm** (a)	**113 nm × 79 nm** (a)	**3 µm × 3 µm**	250 nm × 160 nm	**110 nm × 60 nm**
	Pulse energy	0.66 mJ (*1) at 5.5 keV (7×10^{11} photons)	(2.25 mJ (*2) at 8.5 keV) (2×10^{12} photons)	(3 mJ (*3) at 8.5 keV) (2×10^{12} photons)	**0.54 mJ** at 6 keV (6×10^{11} photons)	~0.5 mJ at 5 keV (5×10^{11} photons)	**0.061 mJ** at 4.05 keV (9×10^{10} photons)
	Photon density (photons μm^{-2} $pulse^{-1}$)	**1.9×10^{12}** (b)	($>8.8 \times 10^{13}$) (*4)	(1×10^{14}) (*4)	**6×10^{10}**	~5×10^{12} (*7)	**$>4 \times 10^{12}$**
**Beamline transmission** (a ratio of pulse energies at focus to source)		(20% (Assumed)) (*5)	(48% (Assumed)) (*6)	(Not shown)	22%	~20%	13%
**Source:**	Size (FWHM)	~70 µm	~60 µm	~60 µm	~50 µm	~50 µm	~80 µm
	Pulse energy	**3.29 mJ**	(4.73 mJ)	-	**2.5 mJ**	~2.5 mJ	**0.47 mJ**
	Photon density (photons μm^{-2} $pulse^{-1}$)	7×10^{8}	(9×10^{8})	-	9×10^{8}	~9×10^{8}	1×10^{8}
**Total gain** (a ratio of photon densities at focus to source)		3×10^{3}	(1×10^{5}) (*4)	-	7×10^{1}	~5×10^{3} (*7)	$>4 \times 10^{4}$

The first and second values in the table cells for the focal lengths, demagnification ratios, and focus sizes are for horizontal and vertical focusing, respectively.
The values in bold were directly characterized experimentally.
(a) Values characterized by wavefront sensors.
(b) Value characterized by CDI measurement of a virus.
(*1) Calculated values using the photon density at the focus characterized by the CDI measurement of a virus and the assumed beamline transmission of 20%. In Supplementary Reference 2, the calculation depends on the assumed beamline transmission: the focus size scales as the square root of the beamline transmission, and the pulse energy is proportional to the beamline transmission. For example, the focus size is given by 117 (369) nm (FWHM) when assuming a beamline transmission of 1% (10%).
(*2) Calculated values using the assumed beamline transmission of 48%.
(*3) Typical LCLS (upstream) pulse energy.
(*4) Calculated values using the beam profile characterized by wavefront sensors and the assumed pulse energy at the focus. The photon density at the focus has not been fully evaluated in CDI configurations.
(*5) Supplementary Reference 2 shows the estimation of focus size with different assumptions on beamline transmission, including the 20% transmission listed here. Beamline transmission is not known.
(*6) Calculated value when assuming the reflectivity of two flat offset mirrors (0.995 × 0.995), the average reflectivity of the K–B mirrors (0.98 × 0.98), and the acceptance loss of the K–B mirrors (0.50).
(*7) Comparison of these calculated values with the corresponding measured values in other columns needs care: the deviation from ideal focusing causes decreases in photon density at the focus and the total gain.

Supplementary Note 1: Numerical simulations of coherent diffraction patterns to estimate spatial resolution for a low-density object.

We have conducted simulations using a Rice Dwarf Virus (RDV) as a model virus to estimate an image resolution with the developed system. As in the previous study[8], the RDV particle was modeled as a homogeneous sphere with a diameter of 71 nm and a mass density of 1.381 g cm^{-3} (Supplementary Figure 1a). For comparison, we also prepared an electron density model of the two bipyramidal AuNPs (Supplementary Figure 1b) based on the reconstructed image. The two models have almost the same number of electrons: 8.2×10^7 for RDV and 8.5×10^7 for AuNPs.

We calculated coherent X-ray diffraction (CXD) patterns of the model objects for two cases where they are placed in a vacuum and solution (Supplementary Figure 2). The incident fluence was set to 9.1×10^{12} photons μm^{-2} $pulse^{-1}$, which was experimentally estimated from the central intensity of the reconstructed CXD pattern. Poisson noise is included in the calculation. The scattering intensities of the model virus without facets are weak at high momentum transfer. In addition, the diffraction intensities of the AuNPs are similar in a vacuum and solution, while the model virus shows weaker diffraction intensities in a solution than in a vacuum.

Supplementary Figure 3 shows the scattering curves of the model virus obtained by circularly averaging the CXD patterns in Supplementary Figure 2a and 2b. The single-shot spatial resolutions estimated solely from the scattering intensities are ~4.8 nm in a vacuum and ~9.3 nm in a solution, when using a threshold of, *e.g.*, five photons per Shannon pixel on the scattering curves (a criterion for single-shot resolution proposed in Supplementary Reference 9; The criterion corresponds to 0.8 photons per 11×11 pixels in our simulation.). It is noted that a much lower threshold of, e.g., 0.16 photons per Shannon pixel (0.04 photons per simulation pixel of Supplementary Reference 10) is used in three-dimensional reconstruction from many CXD patterns of reproducible samples[10]. In practice, it is necessary to consider parasitic scattering noise contained in the experimental data.

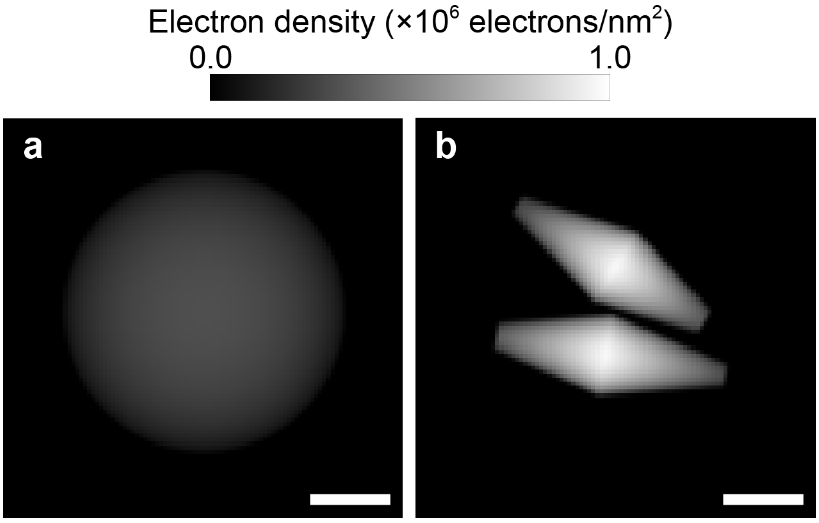

Supplementary Figure 1 | Electron density models. (**a**) Rice Dwarf Virus (RDV). (**b**) Two bipyramidal AuNPs. The scale bars are 20 nm.

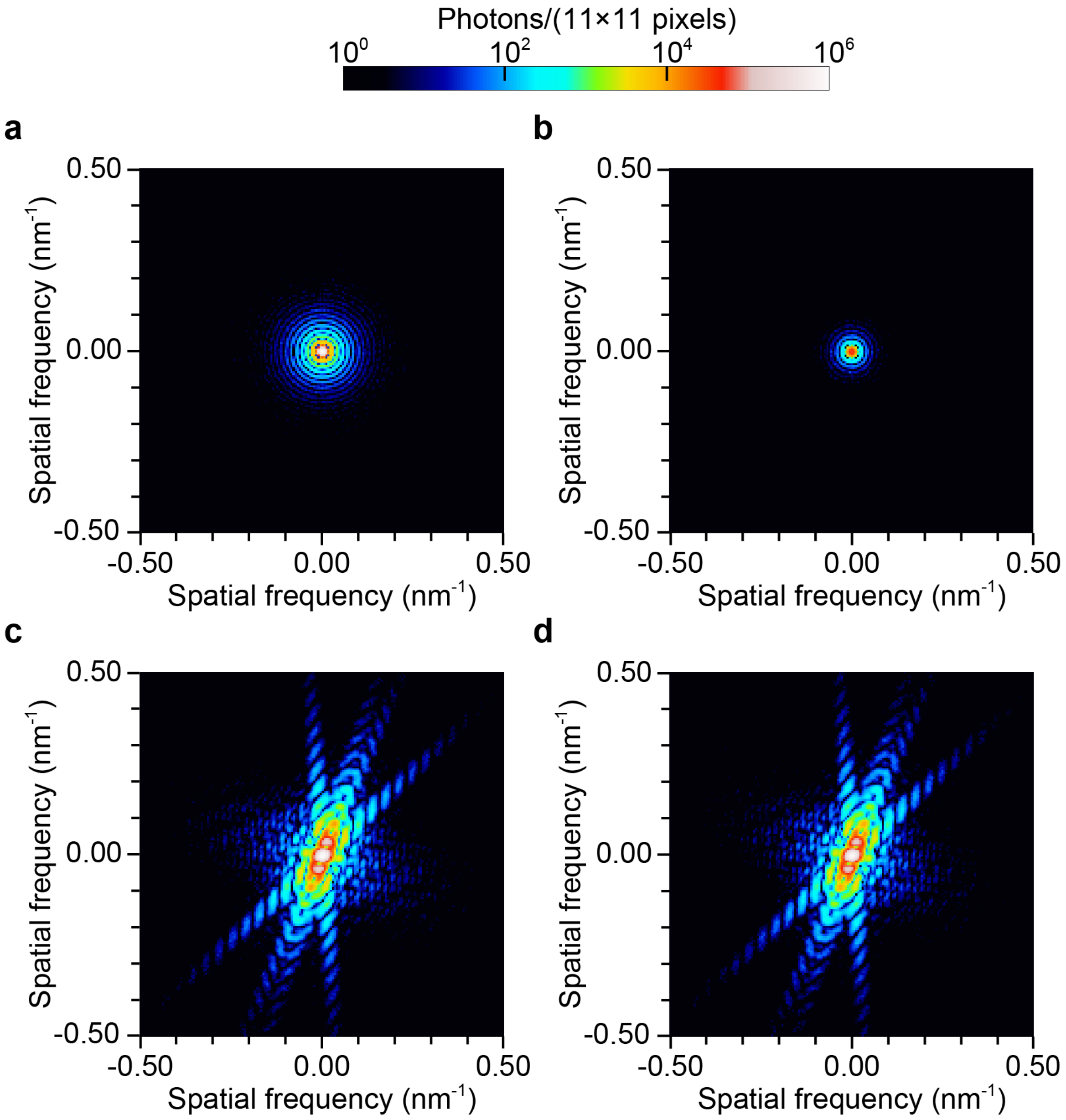

Supplementary Figure 2 | Coherent X-ray diffraction patterns. These are calculated with an incident fluence of 9.1×10^{12} photons μm^{-2} $pulse^{-1}$. (**a**) and (**b**) Diffraction patterns of the model virus (Supplementary Figure 1a). (**c**) and (**d**) Diffraction patterns of the model AuNPs (Supplementary Figure 1b). The sample objects are placed in a vacuum (**a** and **c**) and in a solution (**b** and **d**).

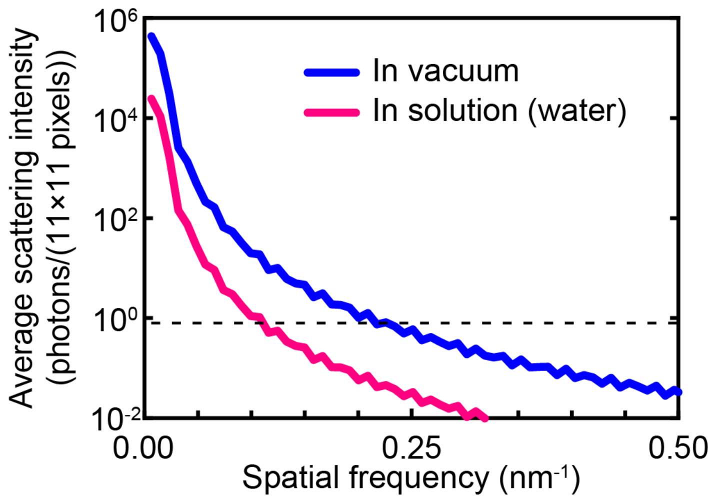

Supplementary Figure 3 | Circularly averaged scattering curves of the model viruses. The horizontal dashed line corresponds to five photons per Shannon pixel, a threshold for single-shot resolution proposed in Supplementary Reference 9.

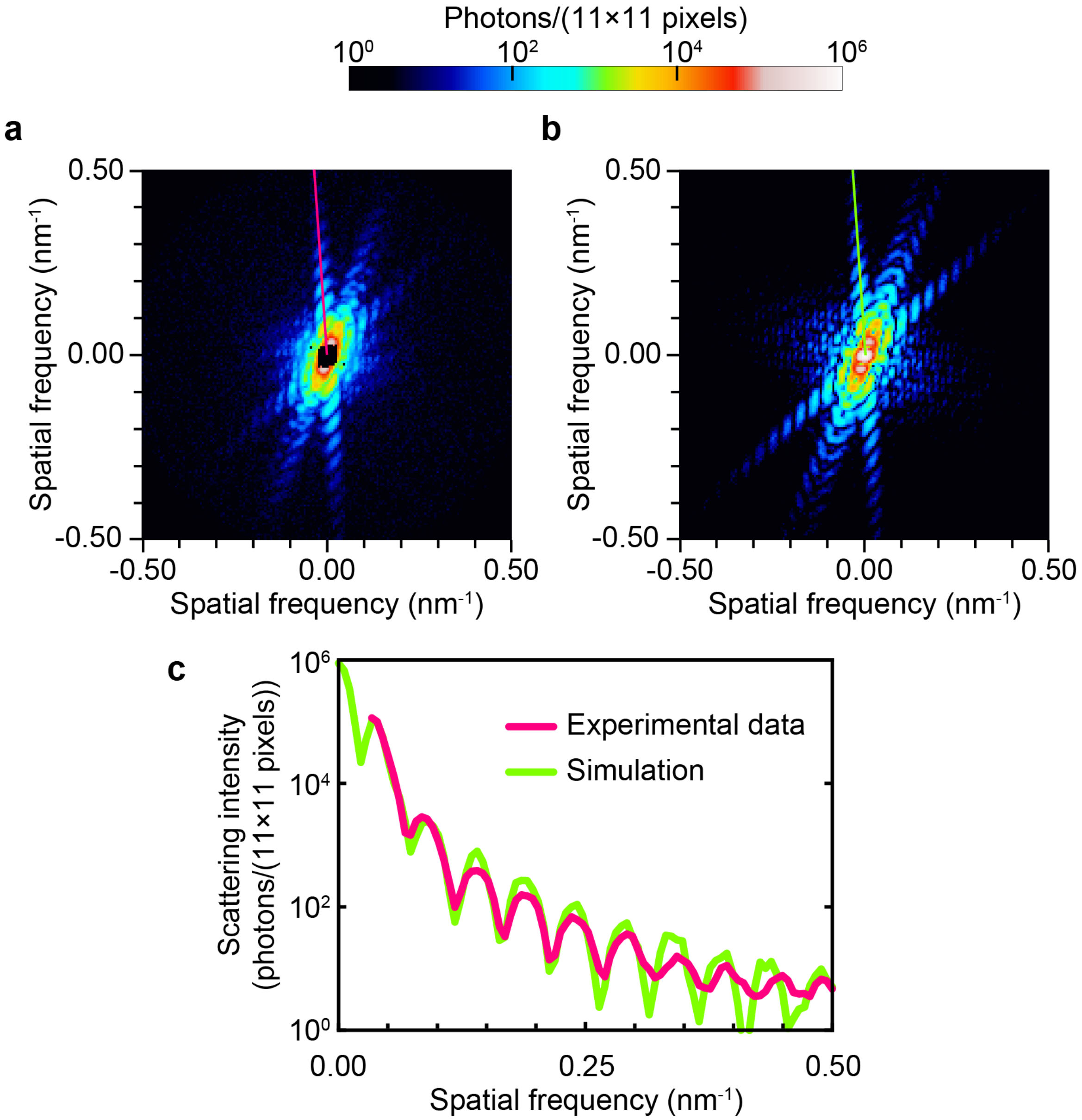

Supplementary Figure 4 | Coherent X-ray diffraction patterns from AuNPs. (**a**) Measured result (replication of Figure 4b of the main text). (**b**) Calculated result (replication of Supplementary Figure 2d). (**c**) Profiles along the lines indicated in **a** and **b**.

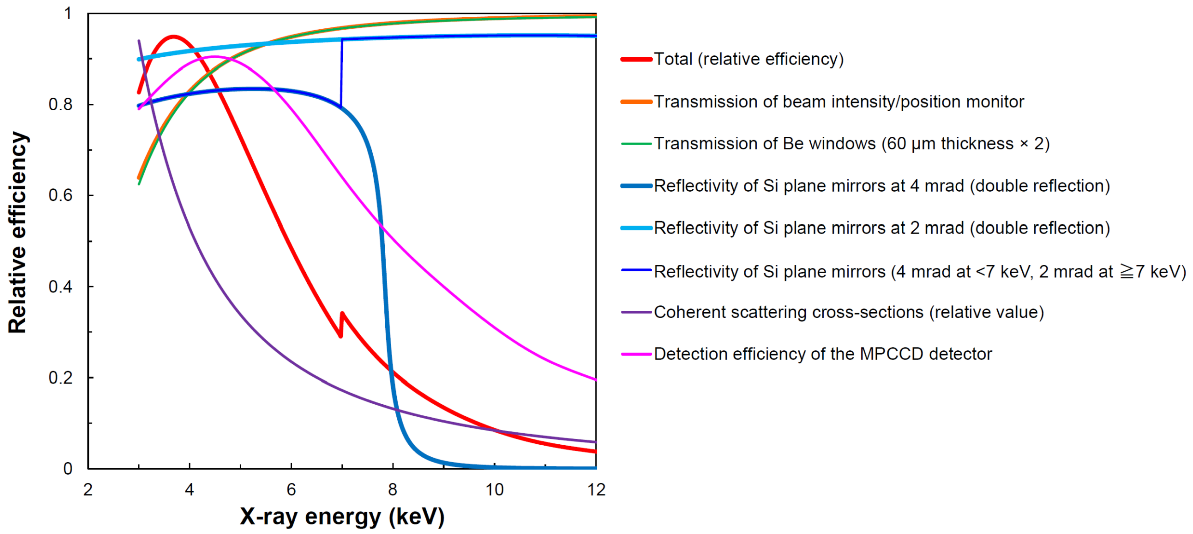

Supplementary Figure 5 | Energy dependencies of efficiency for various factors. Total efficiency has a maximum at around 4 keV. Transmission curves of beam intensity/position monitor and Be windows overlap each other. Coherent scattering cross-sections are assumed to be proportional to the X-ray wavelength squared[11]. Diffraction signals from objects are collected by the multiport charge-coupled device (MPCCD) detector system[12].

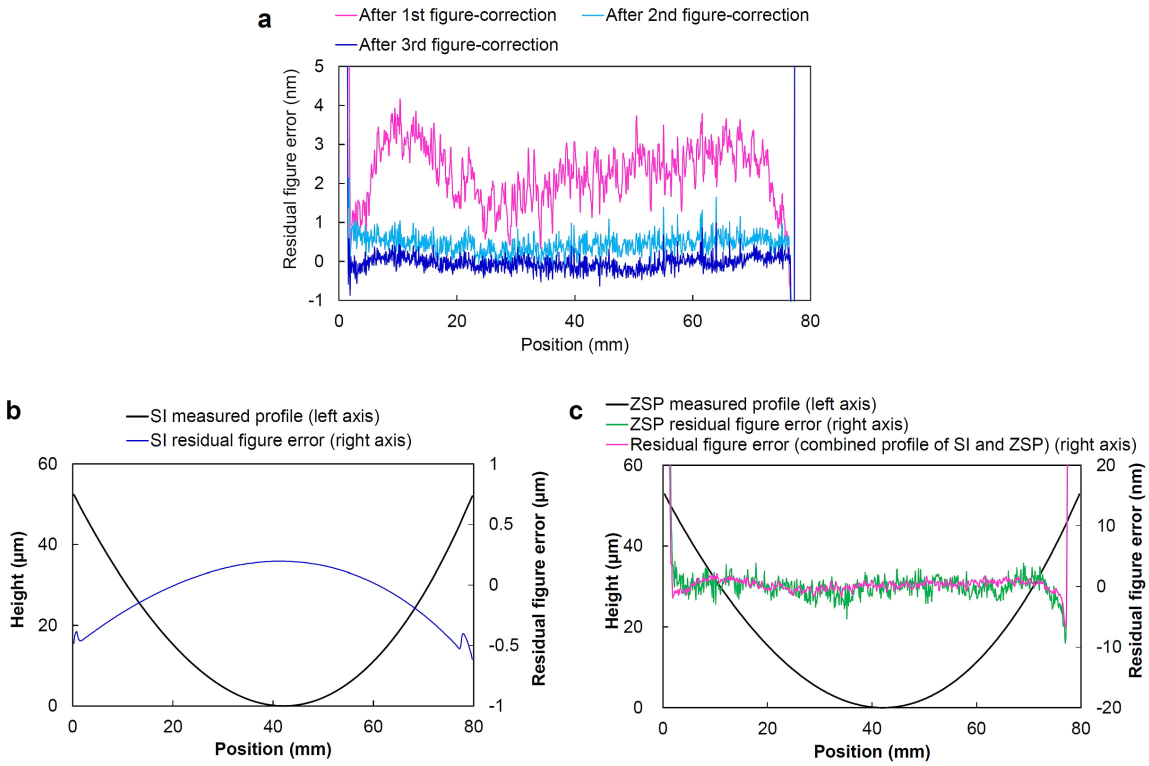

Supplementary Figure 6 | Measurement results of stitching interferometer (SI) and scanning probe profilometer (zero-method scanning-probe profilometer (ZSP)). The substrate surface of the horizontal focusing mirror was measured in the fabrication process using computer-controlled figure-corrections. (**a**) Residual figure error profiles of the last three figure-correction processes. The 3rd figure-correction was the final figuring process. (**b**) and (**c**) Measured profiles with the SI and ZSP, respectively, after the 1st figure-correction in **a**. 'Residual figure error (combined profile of SI and ZSP)' in **c** is replication of the residual figure error profile 'After 1st figure-correction' in **a**. The two-dimensional profile of a residual figure error after the final figure-correction is shown in Figure 1(c) of the main text.

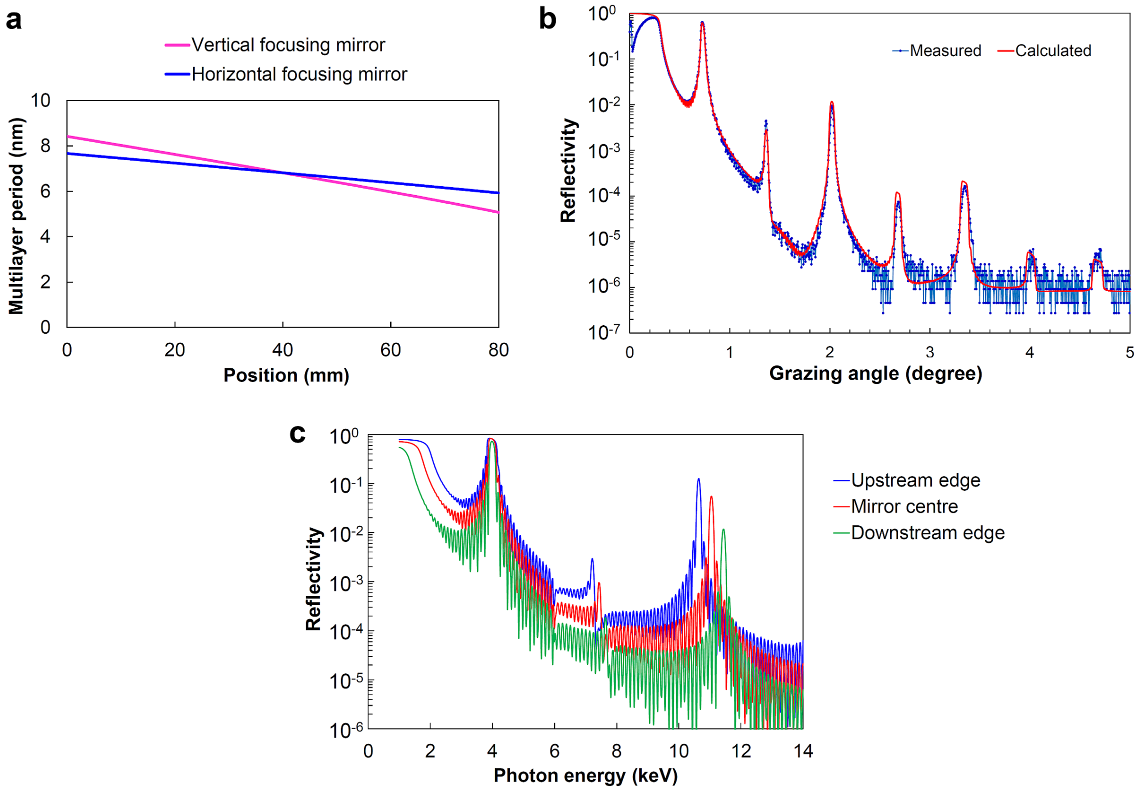

Supplementary Figure 7 | Lateral profiles and reflectivity of $(Cr/C)_{30}$ multilayer. (**a**) Lateral profiles of multilayer period. (**b**) Reflectivity of $(Cr/C)_{30}$ multilayer measured and calculated at a photon energy of 8.048 keV (Cu Kα). The laterally graded multilayer was coated on a flat Si wafer at the same time as the vertical focusing mirror. The calculated reflectivity was obtained by the multilayer model as follows: (1) Material structure is $(Cr/C)_{30}$ on a Si substrate. (2) Ratio of (Cr layer thickness)/(Period) is 0.48. (3) Only the topmost C layer is assumed to have a thickness of 4.3 nm. (4) Density of C and Cr are 2.5 and 7.15 g cm^{-3}, respectively. (5) Interdiffusion/roughness is 0.34 nm (r.m.s.). Because the illumination width at this reflectivity measurement was 3 mm in the longitudinal direction, the reflectivities for a multilayer period from 6.57 to 6.69 nm are averaged. This multilayer has a lateral graded structure in the longitudinal direction having the lateral profile as shown in **a**. The reflectivity was measured in the orthogonal geometry (in the short axis direction) at 8.048 keV (Cu Kα). (**c**) Calculated reflectivity of the vertical focusing mirror at an interdiffusion/roughness of 0.3 nm (r.m.s.). Due to the index of refraction of the multilayer materials used, the third-order Bragg peak is not exactly at a photon energy of 12 keV. Therefore, the third-order harmonic from the undulator source with a photon energy of 12 keV does not fulfill the Bragg condition.

Supplementary References

1. Liang, M. *et al.* The Coherent X-ray Imaging instrument at the Linac Coherent Light Source. *J. Synchrotron Radiat.* **22**, 514–519 (2015).
2. Daurer, B. J. *et al.* Experimental strategies for imaging bioparticles with femtosecond hard X-ray pulses. *IUCrJ* **4**, 251–262 (2017).
3. Seaberg, M. H. *et al.* Nanofocus characterization at the Coherent X-ray Imaging instrument using 2D single grating interferometry. *Proc. SPIE* **11038**, 110380L (2019).
4. Makita, M. *et al.* Double grating shearing interferometry for X-ray free-electron laser beams. *Optica* **7** 404–409 (2020).
5. Bean, R. J., Aquila, A., Samoylova, L. & Mancuso, A. P. Design of the mirror optical systems for coherent diffractive imaging at the SPB/SFX instrument of the European XFEL. *J. Opt.* **18**, 074011 (2016).
6. Ayyer, K. *et al.* 3D diffractive imaging of nanoparticle ensembles using an x-ray laser. *Optica* **8**, 15–23 (2021).
7. Yoon, C. H. *et al.* A comprehensive simulation framework for imaging single particles and biomolecules at the European X-ray Free-Electron Laser. *Sci. Rep.* **6**, 24791 (2016).
8. Munke, A. *et al.* Coherent diffraction of single Rice Dwarf virus particles using hard X-rays at the Linac Coherent Light Source. *Sci. Data* **3**, 160064 (2016).
9. Mancuso, A. P., Yefanov, O. M. & Vartanyants, I. A. Coherent diffractive imaging of biological samples at synchrotron and free electron laser facilities. *J. Biotechnol.* **149**, 229–237 (2010).
10. Fung, R., Shneerson, V., Saldin, D. K. & Ourmazd, A. Structure from fleeting illumination of faint spinning objects in flight. *Nat. Phys.* **5,** 64–67 (2009).
11. Shen, Q., Bazarov, I. & Thibault, P. Diffractive imaging of nonperiodic materials with future coherent X-ray sources. *J. Synchrotron Radiat.* **11**, 432–438 (2004).
12. Kameshima, T. *et al.* Development of an X-ray pixel detector with multi-port charge-coupled device for X-ray free-electron laser experiments. *Rev. Sci. Instrum.* **85**, 033110 (2014).
